# RAB7A-enriched extracellular vesicles drive early-metastasis and immune evasion in triple-negative breast cancer

**DOI:** 10.7150/ijbs.138205

**Published:** 2026-09-18

**Authors:** Hanyi Zhong, Han Wang, Weiqi Gao, Ziling Zhou, Shoutang Wang, Ou Huang, Renhong Huang, Zheng Wang, Kunwei Shen

**Affiliations:** 1 Department of General Surgery, Comprehensive Breast Health Center, Ruijin Hospital, Shanghai Jiao Tong University School of Medicine, 197 Ruijin Second Road, Shanghai 200025, China.; 2 School of Biomedical Sciences, Li Ka Shing Faculty of Medicine, University of Hong Kong, Hong Kong 999077, China.

**Keywords:** small extracellular vesicle, triple-negative breast cancer, RAB7A, anti-PD-1, early metastasis, combined therapy

## Abstract

Early-metastatic triple-negative breast cancer (TNBC) is an aggressive subtype of breast cancer and responds poorly to immunotherapy. However, mechanisms of metastasis or immune evasion remain incompletely understood. This study demonstrates that tumor-derived small extracellular vesicles (sEV) from a clinical cohort of 16 TNBC patients promote cytoskeletal remodeling, tumor growth, and lung metastasis. Label-free quantitative proteomic analysis revealed that RAB7A is enriched in sEVs from early-metastatic patients, whereas other endolysosomal proteins were not markedly altered. Mechanistically, sEV-delivered RAB7A activated the MAPK/ERK signaling pathway, thereby promoting paxillin phosphorylation, F-actin polymerization, and enhanced cell motility. Concurrently, RAB7A upregulated c-MYC expression which bound to the CD274 promoter and increased PD-L1 expression, contributing to immune evasion. Importantly, combined treatment with the RAB7A inhibitor CID1067700 and anti-PD-1 suppressed both tumor growth and metastasis, and enhanced intratumoral infiltration of CD8⁺ T cell. Collectively, this study found that the sEV/RAB7A/ERK axis promotes early metastasis and immune evasion in TNBC, and propose that targeting RAB7A-enriched sEV plus PD-1 blockade represents a mechanistically rational therapeutic strategy in aggressive TNBC.

## Introduction

Breast cancer is one of the most common malignancies affecting women globally 1. Triple-negative breast cancer (TNBC) is characterized by the lack of hormone receptor and HER2 expression 2. In contrast to other subtypes, TNBC exhibits greater invasiveness, resulting in higher rates of locoregional relapse and early distant metastasis. Owing to the lack of established therapeutic targets, TNBC treatment lacks the precision offered by endocrine or anti-HER2 therapies available for other subtypes 3,4. With the breakthrough clinical application of immune checkpoint inhibitors (ICIs), immunotherapy has thought to be a highly promising direction in TNBC management 5-7. In particular, programmed cell death protein-1 (PD-1) antibodies are of great interest 8.

Early-metastasis TNBC is typically defined as disease metastasis or progression within 12 months of completing adjuvant chemotherapy or surgery 9. Real-world data indicate that early-metastatic TNBC exhibits intrinsic or acquired resistance to adjuvant chemotherapy. The majority of early-relapse TNBC patients have a DFI of < 6 months and a median overall survival (OS) of < 12 months, signifying a dire prognosis 10. Most critically, this subset of patients with the most aggressive disease is precisely the one that appears insensitive to current ICI-based immunotherapy 11,12. Clinical data from the KEYNOTE-355 and IMpassion132 trials demonstrate that early-metastatic TNBC patients exhibit inferior responses with anti-PD-1/PD-L1 therapy and has failed to significantly improve survival outcomes 13-15. However, the molecular determinants that distinguish early-metastatic TNBC from other TNBC courses remain poorly defined. The underlying mechanisms of this therapeutic resistance remain unclear but suggest that early-metastasis TNBC may foster a highly immunosuppressive or dysfunctional tumor immune microenvironment (TIME)^16^. Therefore, elucidating the unique biological underpinnings of early-relapse TNBC, particularly the key mechanisms mediating immunotherapy resistance, and identifying more precise therapeutic targets and effective combination strategies are scientific imperatives.

In recent years, extracellular vesicles (EVs), especially small EVs (sEVs, commonly referring to exosomes with a diameter < 200 nm), have garnered extensive attention as important vehicle of communication within tumor cells in cancer research 17. Secreted by cells and enveloped by a lipid bilayer, sEVs carry and deliver cargoes. sEVs are loaded with proteins and nucleic acids to recipient cells, thereby remotely modulate their function and fate 18. In oncology, sEVs have been proven to play central roles in mediating tumor growth, angiogenesis, pre-metastatic niche formation, chemoresistance, and immune regulation 19,20. In TNBC, tumor-derived sEVs can promote metastasis, induce epithelial-mesenchymal transition (EMT), and suppress anti-tumor immune responses 21. Moreover, because sEVs are stably present in body fluids like blood and mirror the molecular signatures of their parent cells, they show immense potential as liquid biopsy biomarkers for the non-invasive diagnosis 22,23, treatment response monitoring, and prognosis prediction of early-metastasis TNBC. Our group previously identified the endoplasmic reticulum-shaping protein reticulin 4 (RTN4) as enriched in plasma EVs from metastatic TNBC patients, and demonstrated that RTN4 promotes EMT and immune evasion through NF-κB activation 24. Although RTN4 holds value as a general prognostic marker for metastatic recurrence, it does not distinguish early from late relapse. Thus, we hypothesize that early-metastatic TNBC may be driven by a distinct, sEV-mediated molecular mechanism that promotes both metastatic dissemination and immunosuppression.

RAB7A, a member of the Rab GTPase family, classically regulates late endosomal/lysosomal trafficking, autophagy, and cytoskeletal reorganization 25. Emerging evidence suggests that RAB7A may possess signaling regulatory capacity beyond its degradative functions. Yet, no studies have investigated whether RAB7A can be delivered via sEV to recipient cells and exert such non-canonical functions 26,27. RAB7A is known to play a role in the development of several cancer types, but its specific role in TNBC, particularly its coupling with sEV functionality in the context of early metastasis and immune modulation, remains entirely unexplored.

In this study, we established a clinical cohort of early-metastasis and metastasis-free TNBC patients. Proteomic analysis of plasma sEVs identify the selective enrichment of RAB7A. Upon uptake, sEV-delivered RAB7A activates ERK/c-MYC, driving cytoskeleton remodeling and PD-L1 upregulation to foster immunosuppression. Thus, combined targeting of sEV-RAB7A and anti-PD-1 may be a promising approach to suppress TNBC metastasis.

## Methods

### Clinical Samples

All participants were recruited from the Comprehensive Breast Health Center, Ruijin Hospital between January 2020 and December 2022. Patients were diagnosed of TNBC breast cancer with a histologically confirm. Immunohistochemistry (IHC) staining of ER and PR is < 1% of positive tumor cells. HER2 IHC scores is of 0 or 1+, or fluorescence in situ hybridization shows no amplification of HER2 gene. Metastatic sEVs(M-sEV) were isolated from the plasma of early-metastatic TNBC patients (disease metastasis within 12 months of completing adjuvant chemotherapy or surgery). Non-metastatic sEVs (NM-sEV) were obtained from those remained metastasis-free. Clinical data, including age, tumor size, and lymph node status, were recorded for all patients. A tissue microarray was constructed from 104 TNBC cases with complete clinical, pathological, and prognostic data to support survival analysis. A freshly resected tumor specimen (2 cm × 2 cm) was used for patient-derived organoids (PDOs).

### Cell Culture

The human TNBC cell lines MDA-MB-231 and BT-549, as well as the murine TNBC cell line 4T1, were utilized in this study. High-glucose Dulbecco's Modified Eagle Medium (DMEM) with 10% fetal bovine serum (FBS) was used for MDA-MB-231 cells culture. BT-549 and 4T1 cells were maintained in Roswell Park Memorial Institute-1640 (RPMI-1640) medium containing 10% FBS, BT-549 medium was supplemented with 10 µg/mL insulin to support optimal cell growth. All cell lines were grown at 37 °C in a humidified atmosphere with 5% CO₂.

### sEV Isolation

sEVs were isolated from cell culture supernatant and patient plasma, respectively. The detailed methods are provided in the Supplementary Methods.

### sEV Analysis and mass spectrum

sEV size distribution and particle number/mL of solution were measured by nanoparticle tracking analysis (NTA). sEV morphology was evaluated by transmission electron microscopy. sEV markers (CD63 and TSG101 as positive markers, Calnexin and Histone H3 as negative markers) were validated by Western blot. Label-free quantitative proteomic analysis of sEV samples was performed based on established methods 28. The mass spectrometry data were processed by MaxQuant software 29. Proteins with differential expression were determined by Student's t-test, applying a significance threshold of p < 0.05.

### sEV Uptake Test

The uptake of sEVs by TNBC cells was visualized using the lipophilic dye PKH26. Specifically, 60 µL sEVs in PBS were mixed with PKH26 in Diluent C (Sigma-Aldrich), incubated for 4 min, and quenched with 0.5% bovine serum albumin (BSA). Labeled sEVs were pelleted and resuspended in 100 µL PBS. TNBC cells were incubated with PKH26-labeled sEVs for 24 hours, rinsed twice, and fixed with 4% formaldehyde. Nuclei were stained with DAPI. The internalization was examined using a fluorescence microscope.

### IHC

Tissue sections derived from mouse tumor samples or human tissue microarrays were fixed and embedded. IHC staining was processed using a standard protocol. Samples were incubated overnight at 4 °C with primary antibody (as detailed in Supplementary Table S1). After washing with PBS, the samples were incubated with HRP-conjugated secondary antibodies for 1 hour. Immunostaining was assessed independently by two pathologists. The H-score system was used for evaluation, based on the proportion of positive tumor cells (0 for < 5%, 1 for 5-25%, 2 for 26-50%, 3 for 51-75%, and 4 for > 75%) and staining intensity. The intensity of immunostaining was graded as follows: 0 (negative), 1 (weak), 2 (moderate), or 3 (strong). The H-score was the results of multiplying the percentage scores and intensity scores.

### Gene Knockdown

RAB7A knockdown was achieved through RNA interference using short hairpin RNAs (shRNA). Three shRNA sequences targeting RAB7A were individually cloned into the pLKO.1 vector. Each pLKO.1-shRNA construct was transfected into 293T cells with psPAX2 and pMD2.G used as packaging plasmids. Viral supernatants were added to target cells for 48 hours to facilitate transduction. A pLKO.1 vector with scrambled shRNA sequence was negative control.

### Western Blot

Protein from whole cells or sEV was collected using RIPA lysis buffer. The resulting lysates were separated via SDS-PAGE and electro-transferred onto PVDF membranes. Blots were blocked, probed with primary antibodies (Supplementary Table S1) overnight at 4 °C, followed by a 1-hour reaction with HRP-conjugated secondary antibodies. Immunoreactive signals were revealed by chemiluminescence detection platform.

### Immunofluorescence (IF)

Sterile glass coverslips were placed into 12-well plates, and 1 × 10⁵ cells were seeded per well. Fix cells with 4% paraformaldehyde for 15 minutes after the treatments and permeabilize for 20 minutes at room temperature. Following blocking, the cells were reacted at 4 °C overnight with primary antibodies, and with appropriate fluorescently conjugated secondary antibodies for 1 hour at 4 °C in the dark. The prepared slides were examined using a confocal microscope.

### Wound Healing Assay

In 6-well plates, cells were plated (4 × 10⁵ cells in each well) and reached 90% confluence. A a linear scratch was made across the surface with a 200 µL pipette tip. Then, cells migrated for 48 hours. Phase-contrast microscopy was used to take images at 0 hours and again at 48 hours. The migration rate was calculated using the width of gap of 0 and 48 hours by ImageJ.

### Cell Migration and Invasion Assays

Migration/invasion assays were performed according to established methods 30. Transwell inserts are with 8 µm pores were employed. Cells were prepared at 2.5 × 10⁵ cells/mL using serum-free medium. For the invasion assay, the upper chamber of the Transwell insert was coated with Matrigel. The lower chamber contained 10% FBS medium. After a 24-hour incubation, cells traversed were fixed and stained with 0.1% crystal violet. These stained cells were observed and counted with light microscope. The number of cells was counted in five randomly fields for each insert, and the values were averaged. Each experiment was carried out in triplicate.

### *In vivo* Assays

Female BALB/c mice (aged 5 weeks, weighing 20-25 g) were employed. All animal experiments received approval from the Animal Ethics Committee. Supplementary Figures S4 and S5 show the detailed study designs, including timelines and group allocations. At the experimental endpoint, orthotopic tumor tissues were collected for subsequent analyses.

### *In vivo* Bioluminescence Imaging

For lung metastasis model, luciferase-labeled tumor cells were injected through tail vein. Mice were randomly allocated to different treatment groups. Metastatic progression was monitored using bioluminescence imaging. Before each imaging session, animals received an intraperitoneal injection of D-luciferin (150 mg/kg). Bioluminescent signals were acquired using an IVIS Spectrum *In vivo* Imaging System. The total photon flux (photons/second) was measured within a defined region of interest (ROI) covering the thoracic area, using Living Image software.

### Bulk RNA Sequencing

Bulk RNA sequencing was carried out by Novogene Co., Ltd. (Shanghai, China). Gene expression differences were analyzed with DEGseq. Genes were considered differentially expressed (DEGs) meeting the criteria of p < 0.05 and |fold change| ≥ 1. Functional annotation and pathway enrichment analysis were carried out using Gene Ontology (GO), Kyoto Encyclopedia of Genes and Genomes (KEGG). Enrichment analyses were performed with Gene Set Variation Analysis (GSVA) and Gene Set Enrichment Analysis (GSEA). CIBERSORT with leukocyte gene signature matrix (LM22) estimated 22 immune cell fractions from transcriptomic data.

### Luciferase Reporter Assay

MDA-MB-231 cells were subjected to co-transfection with the PGL4-PDL1 promoter firefly luciferase reporter construct along with the RL-TK Renilla luciferase control plasmid using a suitable transfection reagent. Luciferase activities were assessed using the Dual-Luciferase Reporter Assay System (Promega).

### Patient-derived organoids (PDOs)

PDOs were generated from surgically resected human TNBC specimens and preserved in a biobank according to previously described protocols 31. The culture system for PDOs employed was as methodologies established in earlier studies 32. For all subsequent experiments, organoids at passages 2-3 were utilized.

### Statistical Analysis

All statistical analyses were processed via GraphPad Prism (version 9.3; GraphPad Software, San Diego, CA). At least three independent replicates were performed for each experiment and all data are presented as mean ± standard deviation. Pairwise comparisons were evaluated by two-tailed Student's t-test. One-way analysis of variance (ANOVA) was adopted for multiple groups. Overall survival (OS) was estimated by the Kaplan-Meier method. Survival curves were compared with log-rank test. The threshold for statistical significance was defined as P < 0.05 for all analyses.

## Results

### sEVs from Early-Metastasis TNBC Promote TNBC Migration, Invasion, and Metastasis

The workflow for the study of sEV proteins associated with early metastasis in TNBC is shown in Fig. 1A. Transmission electron microscopy confirmed the characteristic cup-shaped morphology of the isolated sEVs from plasma of TNBC patients (Fig. 1B). Particle size analysis indicated that the majority of sEVs fell within the expected 50-200 nm diameter range (Fig. 1C). Western blot analysis confirmed the presence of common sEV markers (CD63, TSG101) and the absence of negative markers (Calnexin, Histone H3) in sEVs from both early-metastatic TNBC (M-sEV) and non-metastasis TNBC (NM-sEV) patients (Fig. 1D).

To investigate functional uptake, plasma-derived sEVs from both patient groups were stained with PKH26. Laser scanning confocal microscopy confirmed that sEVs from both early-metastatic TNBC and non-metastatic TNBC patients were efficiently internalized by MDA-MB-231 TNBC cells (Fig. S1A).

We next assessed the functional impact of these patient-derived sEVs on TNBC cell behavior. Human TNBC cells were treated with PBS (as control), NM-sEV or M-sEV for 24 hours. Subsequent migration and invasion assays revealed that treatment with M-sEV significantly enhanced the migratory and invasive capabilities of both cell lines compared to the PBS or NM-sEV treatment (Fig. 1E-G, Fig. S1B-C).

The pro-metastatic role of sEV from early-metastasis TNBC was further validated *In vivo*. In a tail vein injection model using MDA-MB-231 cells in NSG mice, treatment with M-sEV promoted the formation of lung metastases compared to the control group (treated with NM-sEV) (Fig. 1J). Similarly, in an orthotopic tumor model, M-sEV significantly accelerated primary tumor growth compared to NM-sEV (Fig. 1H-I).

Consistent with a role in facilitating cell motility, analysis of tumor tissues showed that treatment with M-sEV led to a marked reorganization of the actin cytoskeleton, characterized by increased F-actin polymerization (marked by Fascin) and adhesion plaque formation (marked by paxillin) (Fig. 1K-L). Collectively, these data indicate that sEVs from early-metastasis TNBC patients promote TNBC cell migration, invasion, metastasis, and cytoskeletal remodeling in vitro and *In vivo*.

### RAB7A Is Enriched in Plasma sEVs from Early-Metastasis TNBC and Is Associated with Poor Prognosis

To elucidate the mechanisms by which sEVs from early-metastasis TNBC promote tumor invasion and metastasis, we performed comparative proteomic analysis of plasma-derived sEVs from the early non-metastasis and early- metastasis cohorts. This analysis identified 6 proteins that were significantly upregulated in the M-sEV group (Fig. 2A-B). Intersection of these candidate proteins with breast cancer proteomic data from the public METABRIC cohort yielded four overlapping proteins expressed in breast tumor tissues: NPLOC4, IPO9, KTN1, and RAB7A. Fig. 2C-F show the enrichment of differentially expressed proteins. Pathway enrichment analysis of differentially expressed proteins in plasma-derived sEVs from early-relapse versus non-metastatic TNBC revealed enrichment of several functional categories, including nucleocytoplasmic transport, protein processing in the endoplasmic reticulum, Ras/MAPK-related signaling, tight junction-associated pathways, and platelet/coagulation-related processes (Fig 2C-D). Reactome (Fig 2E) and GO analyses (Fig 2F) also identified host-response and defense-related terms. These results suggest that early-relapse TNBC is associated with a distinct circulating sEV proteomic landscape involving altered intracellular trafficking and secretory pathways, together with signaling modules relevant to cell structural dynamics and metastatic progression.

We next evaluated the prognostic significance of these four proteins using overall survival (OS) data from the METABRIC cohort. Kaplan-Meier survival analysis revealed distinct associations. While NPLOC4 and IPO9 expression showed no significant correlation with patient survival (Fig. S2A-B), higher expression of KTN1 was associated with longer OS (Figure S2C). In striking contrast, elevated expression of RAB7A was strongly correlated with a significantly poorer prognosis (p < 0.01) (Figure 2G).

Notably, RAB7A was the only late endosomal marker selectively elevated in M-sEV among the differentially enriched proteins. This selective enrichment suggests that the elevation of RAB7A in M-sEVs is not simply a consequence of endolysosomal leakage.

To assess the clinical relevance of RAB7A in TNBC tissues, the clinical cohort was stratified into high and low RAB7A groups based on IHC scoring. Survival analysis showed that patients with high RAB7A expression had a significantly worse prognosis (Fig. 2H-I). Consistent with its role in aggressive disease, high RAB7A expression was associated with more advanced tumor (T), nodal (N) and metastasis (M) status (Fig. 2K).

Based on this concordance between our proteomic discovery and clinical validation, we focused validation on RAB7A. ELISA verified that RAB7A protein levels were significantly higher in plasma sEVs from patients with early-metastatic TNBC (Fig. 2J). Furthermore, western blot analysis confirmed the significant enrichment of RAB7A within M-sEV compared to NM-sEV (Fig. S2D).

In the TCGA-BRCA cohort, we found that the enrichment score of CD8 T cells and other immune cells was significantly higher in the RAB7A low-expression group (P < 0.001). This suggests that high RAB7A expression is associated with immunosuppressive TME (Fig. 2L).

These results demonstrate that RAB7A is selectively enriched in plasma sEVs from early-metastasis TNBC patients. Its high expression in tumor tissues is strongly associated with unfavorable clinicopathological features, and poor patient survival, nominating RAB7A as a key molecule linked to aggressive TNBC metastasis.

### RAB7A Within sEV Promotes TNBC Progression, Cytoskeletal Remodeling, and PD-L1 Expression i*n vitro*

To examine the role of RAB7A carried by sEVs in TNBC, we first generated TNBC cell lines with stable knockdown of RAB7A (sh-RAB7A) and a control line (sh-NC). sEVs were then collected from the culture supernatants of these cells using ultracentrifugation. We obtained two sEV populations which one is enriched in RAB7A (RAB7A^High^-sEV) and the other with low RAB7A levels (RAB7A^Low^-sEV). Western blot analysis confirmed the difference in RAB7A content between these sEV groups (Fig. S3A).

We next tested how these sEVs affect TNBC malignant phenotype. When TNBC cells were treated with RAB7A^High^-sEVs, they showed a greater ability to migrate and invade compared to cells treated with PBS or RAB7A^Low^-sEVs (Fig. 3A-D). IF staining showed that cells treated with RAB7A^High^-sEV underwent changes in cytoskeleton. These cells displayed more polymerized F-actin (marked by phalloidin) and phospho-paxillin, forming more pseudopodia, which are features of mobile and invasive cells (Fig. 3E-F).

We also examined whether RAB7A^High^-sEVs induced EMT process which is linked to cytoskeletal remodeling and metastatic capacity. Western blotting (Fig. 3H) and IF (Fig. 3G) analyses in two TNBC cell lines showed that treatment with RAB7A^High^-sEVs markedly reduced the expression of the E-cadherin, while increasing the levels of the N-cadherin and vimentin, compared with PBS or RAB7A^Low^-sEV treatment. These findings indicate that RAB7A^High^-sEVs promote EMT in TNBC cells.

### RAB7A Within sEV Promotes TNBC Progression, Cytoskeletal Remodeling, and PD-L1 Expression *In vivo*

We also tested these effects in living models. We established a lung metastasis model by tail vein injection of TNBC cells. Animals that received RAB7A^High^-sEV developed more metastatic nodules in their lungs than the control groups (Fig. 4A). In an orthotopic tumor model where tumors grow in the mammary fat pad, treatment with RAB7A^High^-sEV made the primary tumors grow faster and larger, which is quantified by their wights and volumes (Fig. 4B-C, Fig. S3B, Fig. 4F-H).

Immunohistochemical and immunofluorescence analysis of tumor tissues from the orthotopic model revealed that treatment with RAB7A^High^-sEV promoted F-actin reorganization within tumors and led to an increasing of PD-L1 expression (Fig. 4D-E, J-K). And Fig. S3C indicated that RAB7A^High^-sEV promotes the EMT process in TNBC. To further investigate the impact of RAB7A^High^-sEV on the immune TME, both IHC and IF analyses consistently showed decreased expression of CD4 and CD8 following RAB7A^High^-sEV treatment, indicating reduced infiltration of effector T cells (Fig. 4J-K). Moreover, ssGSEA revealed a significantly elevated CD8^+^ T cell exhaustion score in the RAB7A^High^-sEV group (Fig. 4I).

Collectively, these findings indicate that sEVs carrying high levels of RAB7A can enhance the movement, invasion, and spread of TNBC cells, remodel the cell's structural framework, and increase PD-L1 levels in tumors, contributing to an immunosuppressive tumor microenvironment.

### sEV-RAB7A activates ERK/c-MYC to upregulate PD-L1 and remodeling cytoskeleton

To elucidate the global transcriptional changes induced by RAB7A depletion, RNA sequencing was performed on TNBC cells with RAB7A knockdown and orthotopic TNBC treated with RAB7A^High^-/RAB7A^Low^-sEV. Differential expression analysis demonstrated widespread gene expression differences between cells with distinct RAB7A expression level (Fig. 5A-B, Fig. S6A, Fig. S6F).

In BT-549 cells, GO analysis of the DEGs demonstrated significant associations with biological processes including MAPK cascade regulation, immune suppression, particularly pathways regulating programmed cell death, extracellular matrix organization, and cytoskeleton remodeling (Fig. 5C). Consistent with this, KEGG pathway analysis revealed a strong enrichment for the MAPK signaling pathway (Fig. 5D). GSEA and GSVA further supported these findings, indicating that higher RAB7A expression was positively correlated with increased activity of MAPK signaling (Fig. 5E-F) and pathways involved in apical junction and EMT (Fig. 5F).

In MDA-MB-231 cells, we observed a similar pattern. GO analysis showed functional enrichments analogous to those in MDA-MB-231 cells (Fig. S6B). KEGG analysis indicated significant involvement of RAB7A-regulated genes in pathways related to cell adhesion and immune response (Fig. S6C). GSEA suggested that RAB7A knockdown was associated with a transcriptional signature corresponding to a lower breast cancer stage grade (Fig. S6E). Furthermore, GSVA demonstrated a coordinated downregulation of multiple cancer-related Hallmark gene sets, including the EMT pathway, upon RAB7A depletion (Fig. S6D). Similar results were obtained from RNA-seq of orthotopic TNBC tumors (Fig. S6F-J).

Building upon transcriptomic evidence from MDA-MB-231 and BT-549 cells linking RAB7A to MAPK signaling, cytoskeletal dynamics, and immune suppression, we next performed in vitro mechanistic validation.

We confirmed that RAB7A delivered via sEVs activates the MAPK/ERK pathway, as evidenced by increased phosphorylation of ERK (Fig. 5G). This activation initiated two downstream cascades. First, activated ERK led to the phosphorylation of paxillin, a regulator of focal adhesions, enhancing cell motility (Fig. 5H). Second, activated ERK signaling upregulated the expression of the transcription factor c-MYC (Fig. 5G). This upregulation was dependent on MAPK/ERK activity, as it was inhibited by the MAPK/ERK pathway inhibitor Trametinib (Fig. 5G-H).

To establish direct transcriptional regulation, chromatin immunoprecipitation (ChIP) assays demonstrated that c-MYC binds to the promoter region of the PD-L1 gene CD274 (Fig. 5I). And luciferase reporter assay further demonstrated that c-MYC activity drives PD-L1 promoter activation (Fig. 5J). Western blotting and IF further confirmed that RAB7A^High^-sEVs regulate PD-L1 expression through the downstream ERK signaling pathway (Fig. 5K-L).

### Combination of RAB7A Inhibitor with Anti-PD-1 Immunotherapy Inhibits TNBC Metastasis

Given the role of RAB7A in modulating the TIME, we evaluated a combination therapy strategy targeting both the RAB7A-driven pathway and ICI. Mouse orthotopic tumor models were randomly assigned to four groups: control, anti-PD-1, CID1067700 (a GTase inhibitor targeting RAB7A), and the combination of anti-PD-1 and CID1067700.

Compared to the control, both monotherapies showed efficacy in reducing tumor growth burden. However, the combination of anti-PD-1 and CID1067700 resulted in significantly greater suppression of primary tumor growth (Fig. 6A, Fig S7A-B) and inhibited lung metastasis (Fig. 6B).

In Fig. 6C, ELISA assays showed that treatment with CID1067700 or anti-PD-1 alone reduced the levels of RAB7A in plasma-sEV from mice. And the combination of CID1067700 and anti-PD-1 further decreased sEV-associated RAB7A levels, suggesting that sEV-associated RAB7A may be involved in the therapeutic effect of the combined regimen.

Analysis of tumor tissues from the combination therapy model indicated enhanced therapeutic effects. The combination treatment more effectively inhibited tumor-promoting cytoskeletal reorganization (as assessed by Fascin and paxillin staining) and increased the CD8+ T cells infiltration compared to either monotherapy (Fig. 6D-G).

To further validate these findings in a human-relevant system, we employed patient-derived TNBC organoids. Organoids were treated with PBS, anti-PD-1, CID1067700, or the combination. The combination treatment exhibited the significant inhibition of organoid growth (Fig. 6H, J). Consistent with the *In vivo* data, analysis of the organoids showed that combined blockade synergistically reduced features of cytoskeletal remodeling and promoted a T-cell-inflamed phenotype (Fig. 6I, K-M).

These results demonstrate that inhibiting the RAB7A/MAPK/ERK axis (using CID1067700) in combination with immunotherapy synergistically suppresses TNBC progression and metastasis. The mechanism involves dual targeting of pro-invasive cytoskeletal changes and the immunosuppressive PD-L1/PD-1 axis, leading to enhanced antitumor efficacy (Fig. 7).

## Discussion

Early-metastatic TNBC is hard to be diagnosed and has highly clinical heterogeneity, leading to significant therapeutic challenges 33. The development of more effective diagnostic and precise therapeutic strategies remains an urgent clinical need 34. In this context, blood-based liquid biopsy has emerged as a powerful tool for non-invasive cancer monitoring. sEVs derived from blood are particularly promising biomarkers due to their rich molecular cargo 35,36.

Our previous research identified that RTN4 is the key molecular within plasma sEVs associated with aggressive TNBC biology. Based on this established platform, we have shifted our focus to address the critical problem of early metastasis. Through analysis of plasma-sEV from TNBC patients with short-interval metastasis, we identified a significant and consistent elevation of RAB7A. This finding suggests that RAB7A may be a differential marker as well as a central molecular potentially driving the aggressive biology underlying rapid disease relapse.

In our study, RAB7A is identified as a key regulator that coordinates a program favorable for tumor progression and associated with metastasis. It is a member of the small GTPase Rab family. Traditionally, it is regarded as a core factor controlling late endosome-lysosome trafficking, endocytic degradation, and autophagy 37. However, its functions extend beyond these classic roles. Recent evidence suggests that RAB7A also contributes to cytoskeleton remodeling and immune regulation. In cytoskeletal dynamics, RAB7A facilitates microtubule-dependent vesicle transport. It also participates in actin network organization, influencing organelle positioning, cell migration, and specialized structural remodeling 38,39. In immune signaling, RAB7A plays a role by regulating the trafficking, degradation, or recycling of immune-related molecule, thereby finely tuning the initiation, duration, and resolution of innate immune responses 40. Its activity can determine whether signaling components are degraded to terminate activation or retained to sustain downstream pathways. Thus, RAB7A bridges fundamental cellular transport with higher-order processes in cellular morphology and immune modulation.

This is accordance with our research results. Our results demonstrate that RAB7A actively involves in shaping both the cancer cells and the extrinsic TME. In functional validation, RAB7A was knocked down in TNBC cell lines. The secreted sEVs from these cells were then compared with those from control cells. It was found that sEVs containing high levels of RAB7A significantly promoted malignant biology of tumors, and the cytoskeletal remodeling in vitro. Consistent with this, in an *In vivo* model, RAB7A^High^-sEVs enhanced the distant metastases and suppressed the infiltration of CD8^+^ T cells. Together, these results clarify the role of RAB7A in regulating both metastasis and the immune tumor microenvironment in TNBC.

For the mechanism, RAB7A activates the MAPK signaling pathway. The activation of ERK phosphorylation initiates two parallel cascades. The first cascade promotes cellular invasion which is the phosphorylated ERK phosphorylates paxillin. This modification triggers reorganization of the actin cytoskeleton. The remodeled cytoskeleton enhances tumor cell motility and invasiveness, and migration into surrounding tissues. This contributes to the formation of metastatic lesions. The second cascade upregulates PD-L1 expression. Activated MAPK/ERK signaling elevates the expression of the transcription factor c-MYC. c-MYC directly stimulates transcriptional activation of the PD-L1 gene. These molecular changes align with the increased PD-L1 expression observed in our functional assays and reveal the mechanism of immune evasion. Together, these findings suggest that targeting the RAB7A/ERK/c-MYC axis may represent a promising therapeutic strategy TNBC.

Therefore, we hypothesis that targeting RAB7A may have potential for treating TNBC. Experimental results revealed that the combined therapy inhibited TNBC progression and reduced lung metastasis. Also, in PDOs and *In vivo* model, we found that the combined treatment synergistically affects the cytoskeletal modulating and the infiltration of CD8^+^T cells. Therefore, targeting RAB7A combined with ICI emerges as a highly promising and clinically actionable strategy for TNBC that clearly deserves in-depth future exploration.

In summary, this study, utilizing a liquid biopsy approach, identifies RAB7A as a potential contributor to early metastasis in TNBC. We have delineated a dual mechanism of action mediated through the MAPK/ERK pathway, which appears to coordinately promote cytoskeletal-driven invasion and c-MYC/PD-L1-mediated immune suppression. These findings partially fill the current gap in mechanism of early-metastatic TNBC. Also, it demonstrates the potential of RAB7A as a therapeutic target. However, this study still has limitations. The reason why RAB7A is enriched in sEVs from early-metastatic TNBC remains unclear. We need further studies to elucidate the mechanisms underlying RAB7A upregulation. This will facilitate the development of more precise therapeutic strategies and more effective diagnostic approaches.

## Conclusion

In conclusion, our study identifies that sEV-RAB7A is a critical mediator of early metastasis and immune evasion in TNBC. We demonstrate that RAB7A-enriched sEVs from early-metastasis TNBC patients actively drive aggressive tumor behavior. Mechanistically, sEV-delivered RAB7A activates the MAPK/ERK pathway in recipient tumor cells, thereby promoting cytoskeletal remodeling and enhancing migratory and invasive capacity. Also, RAB7A induces c-MYC-mediated PD-L1 transcription to support immune escape. Importantly, inhibition of the RAB7A pathway in combination with anti-PD-1 therapy produced antitumor and antimetastatic effects in mouse models and patient-derived organoids. Collectively, this work establishes the sEV/RAB7A/ERK axis as a driver of TNBC progression and immunotherapy resistance. It provides a strong rationale for developing RAB7A-targeted combination strategies to improve outcomes in patients with aggressive TNBC.

## Supplementary Material

Supplementary figures, tables, and methods.

## Figures and Tables

**Figure 1:**
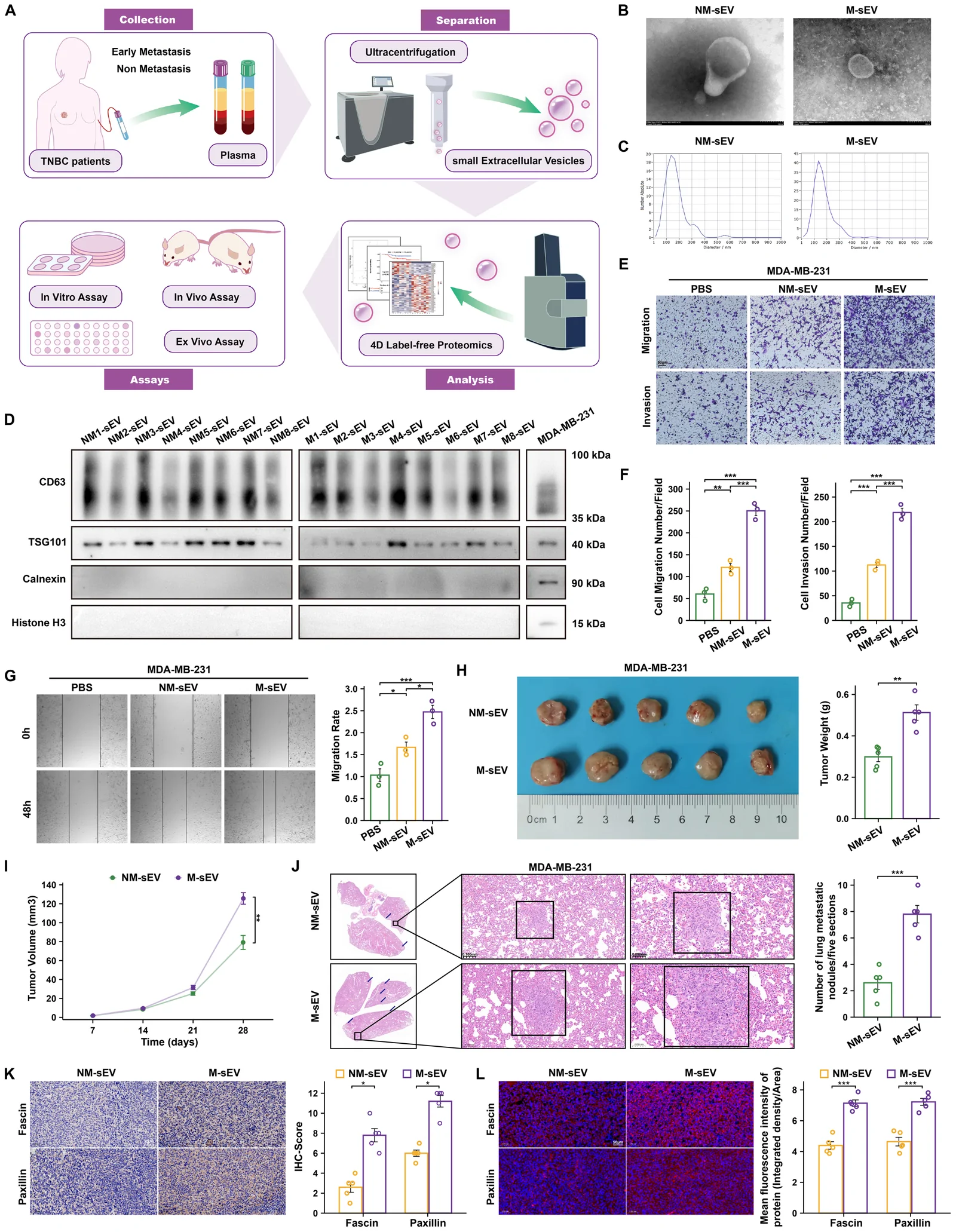
** Early metastasis TNBC sEVs promote TNBC migration, invasion, and metastasis.** (A) Schematic illustration of the experimental workflow, including plasma sEV isolation from patients with non-metastatic (NM) or metastatic (M) TNBC, proteomic profiling, biomarker identification and validation, and functional investigations. (B) Representative transmission electron microscopy images of plasma-derived sEVs. (C) Particle size distribution of plasma-derived sEVs. (D) Western blot analysis of sEV-positive markers (CD63 and TSG101) and negative markers (Calnexin and Histone H3) in sEVs isolated from TNBC patient plasma and MDA-MB-231 cell culture supernatants. (E-G) Representative images and quantitative analyses of Transwell migration/invasion assays (E-F) and wound-healing assays (G) in MDA-MB-231 cells treated with PBS, NM-sEVs, or M-sEVs. (H) Representative images and tumor weights of orthotopic tumors from NSG mice treated with NM-sEVs or M-sEVs. (I) Tumor growth curves of NSG mice in the indicated groups. (J) Representative hematoxylin and eosin (HE)-stained lung sections and quantification of metastatic nodules in the tail-vein metastasis model. (K-L) Representative images and quantitative analyses of cytoskeleton-related markers in tumors from NSG mice, as assessed by immunohistochemistry (K) and immunofluorescence staining (L). *P < 0.05, **P < 0.01, ***P < 0.001. IF: Immunofluorescence; IHC: Immunohistochemistry; M-sEV: Metastatic small extracellular vesicles; NM-sEV: Non-metastatic small extracellular vesicles; NSG: NOD scid gamma; PBS: phosphate buffer saline; TNBC: Triple-negative breast cancer.

**Figure 2:**
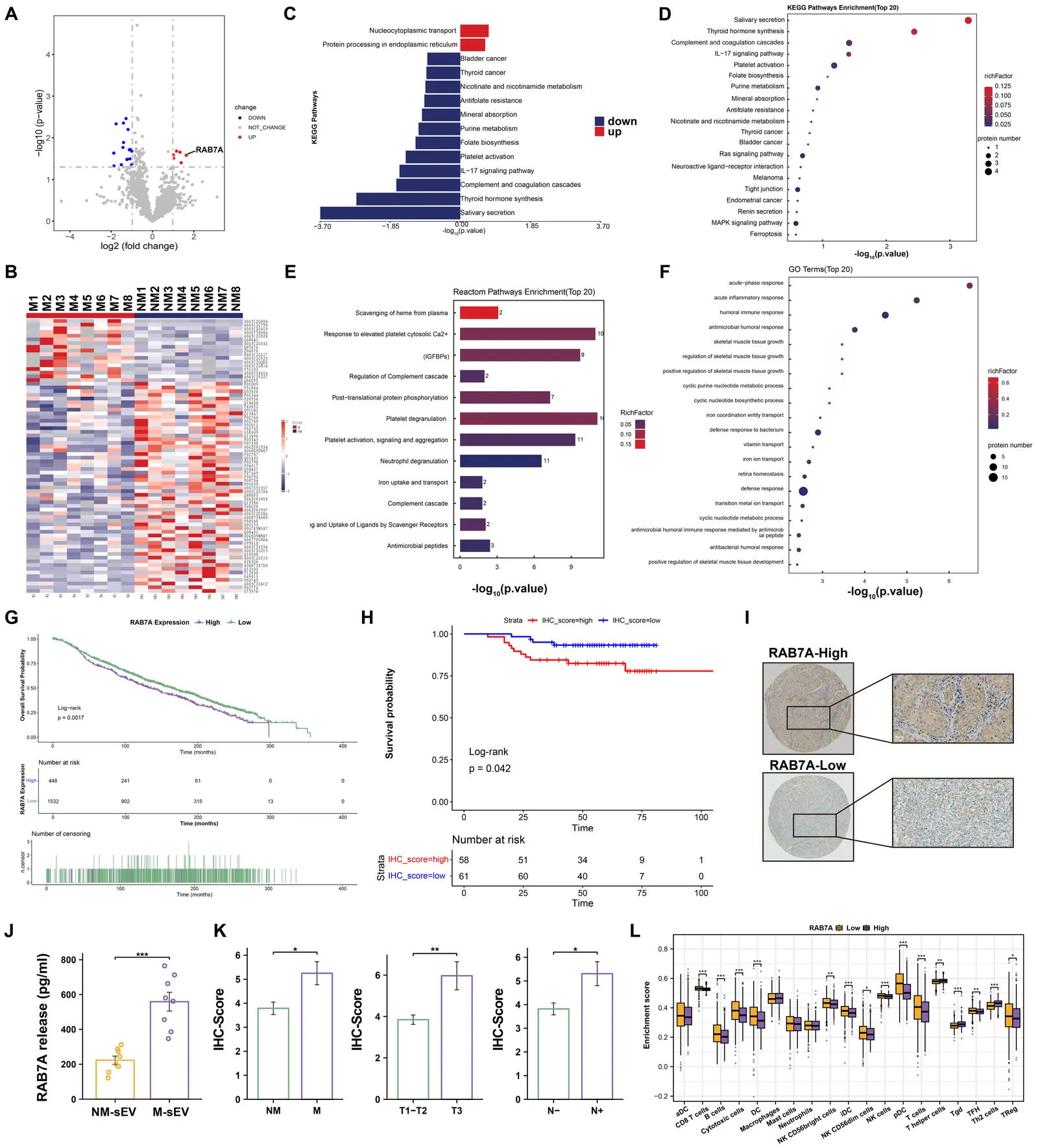
** RAB7A is enriched in plasma sEVs from patients with early-metastatic TNBC and is associated with poor prognosis.** (A) Volcano plot showing differentially expressed proteins in plasma-derived sEVs from patients with metastatic (M) versus non-metastatic (NM) TNBC. (B) Heatmap of differentially expressed proteins between the M and NM groups. (C-F) Functional enrichment analyses of differentially expressed proteins, including KEGG pathway enrichment shown as a bar plot (C) and bubble plot (D), Reactome pathway enrichment analysis (E), and Gene Ontology (GO) term enrichment analysis (F). (G) Kaplan-Meier analysis of overall survival in the TNBC cohort stratified by RAB7A expression. (H) Kaplan-Meier survival analysis of patients with TNBC stratified according to RAB7A IHC score. (I) Representative IHC images of RAB7A expression in TNBC tumor tissues with high or low RAB7A expression. (J) ELISA quantification of plasma sEV-associated RAB7A in the NM-sEV and M-sEV groups. (K) Association of RAB7A IHC scores with metastatic status, primary tumor stage, and lymph-node status in TNBC tissues. (L) The immune-cell enrichment scores in TCGA-BRCA samples by ssGSEA according to RAB7A expression. *P < 0.05, **P < 0.01, ***P < 0.001. ELISA: Enzyme Linked Immunosorbent Assay; IHC: Immunohistochemistry; TNBC: Triple-negative breast cancer.

**Figure 3:**
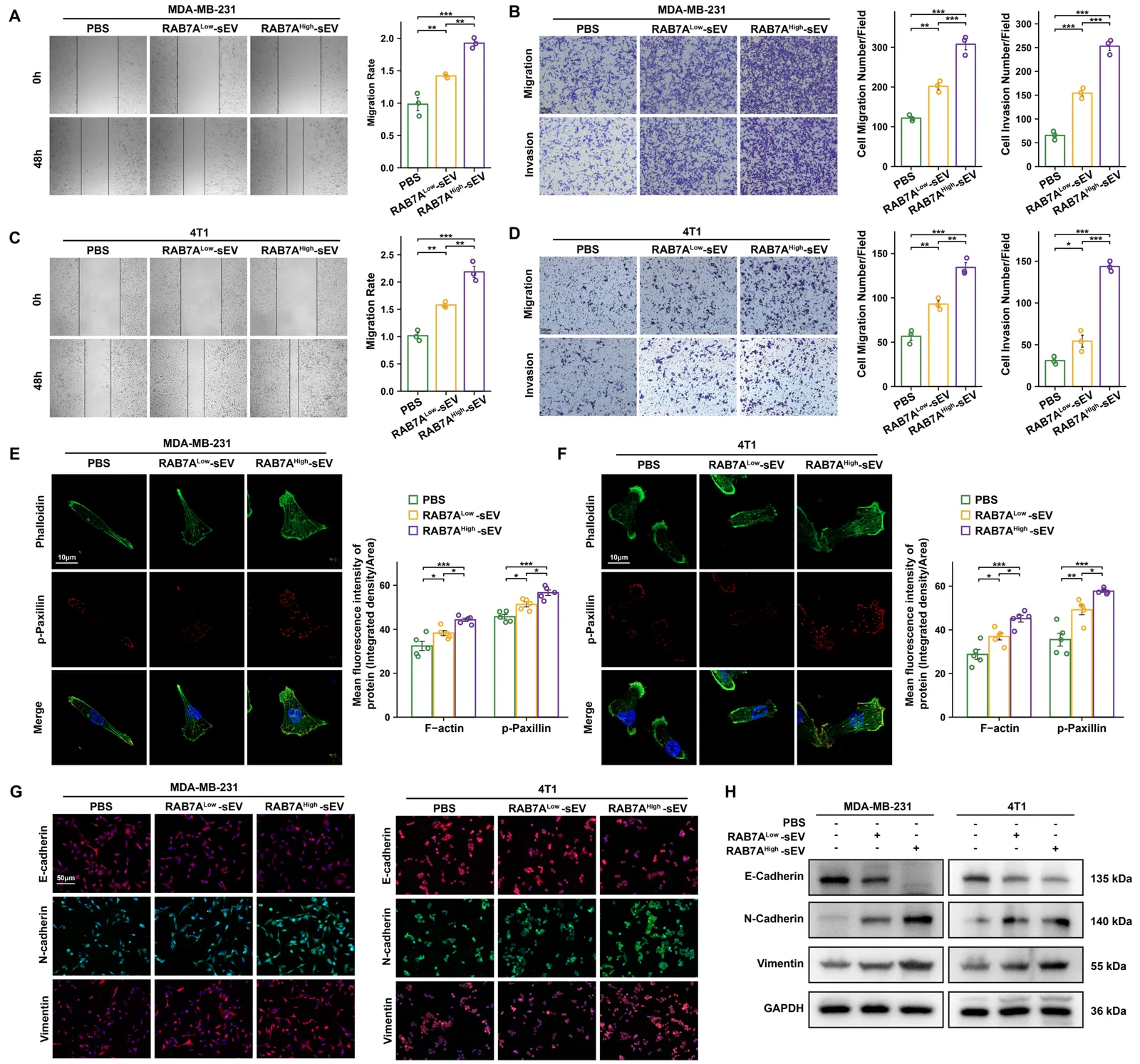
** RAB7A^High^-sEV promotes TNBC cell motility, cytoskeletal remodeling and EMT in vitro.** (A, C) Representative images and quantification of wound-healing assays in MDA-MB-231 (A) and 4T1 (C) cells treated with PBS, RAB7A^Low^-sEV, or RAB7A^High^-sEV. (B, D) Representative images and quantification of Transwell migration and invasion assays in MDA-MB-231 (B) and 4T1 (D) cells following the indicated treatments. (E, F) Representative immunofluorescence images and quantification of F-actin (phalloidin, green) and phosphorylated paxillin (p-paxillin, red) in MDA-MB-231 (E) and 4T1 (F) cells. Nuclei were counterstained with DAPI (blue). (G) Immunofluorescence staining of EMT markers, including E-cadherin, N-cadherin, and vimentin, in MDA-MB-231 and 4T1 cells treated as indicated. (H) Western blot of E-cadherin, N-cadherin, and vimentin expression in MDA-MB-231 and 4T1 cells after treatment with PBS, RAB7A^Low^-sEV or RAB7A^High^-sEV. GAPDH was used as the loading control. *P < 0.05, **P < 0.01, ***P < 0.001. EMT: epithelial-mesenchymal transition; PBS: phosphate buffer saline.

**Figure 4:**
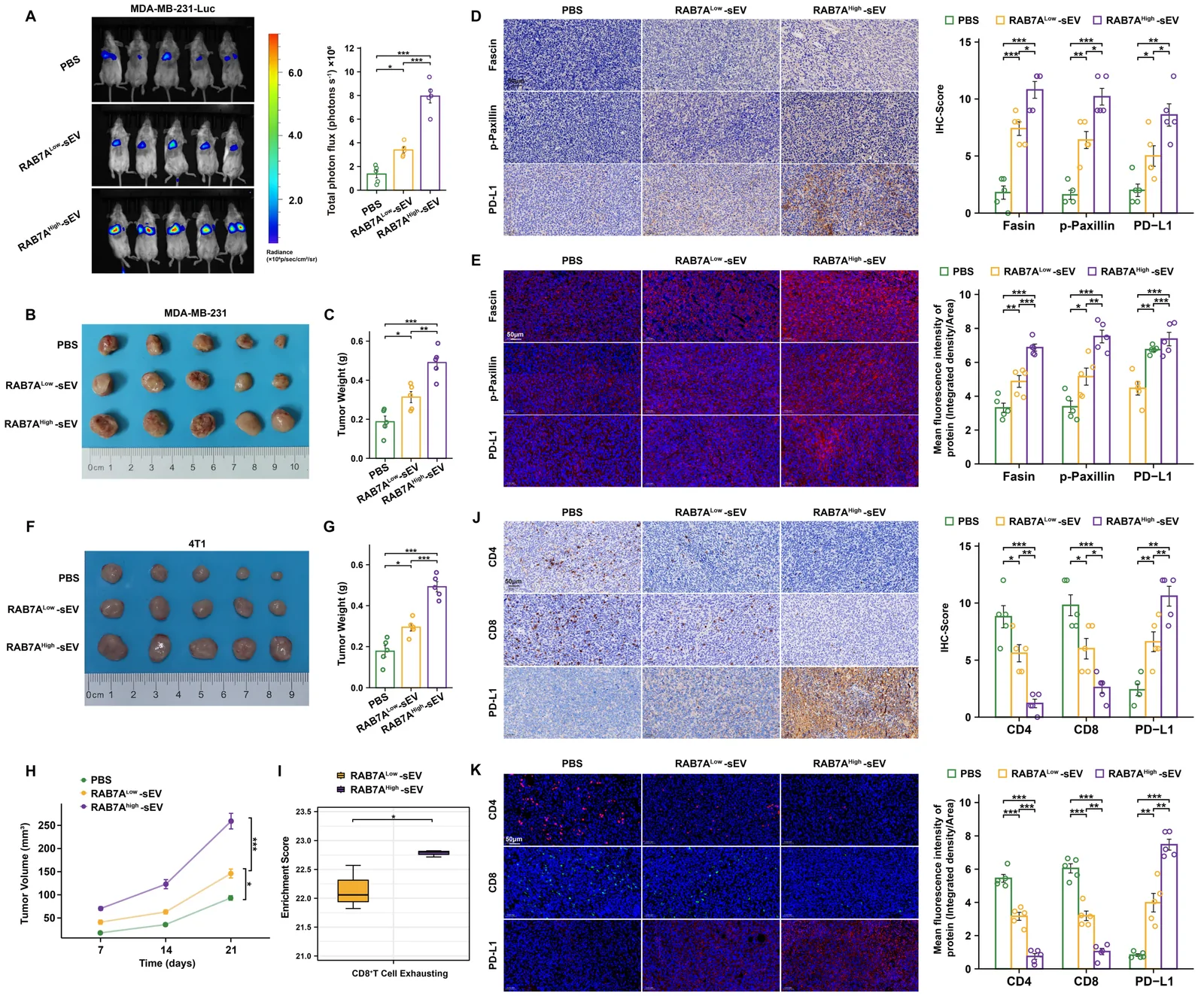
** RAB7A^High^-sEV promotes TNBC cytoskeletal remodeling, tumor metastasis, and an immunosuppressive tumor microenvironment *In vivo*.** (A) Representative bioluminescence images and quantification of total photon flux in mice injected intravenously with MDA-MB-231-Luc cells following treatment with PBS, RAB7A^Low^-sEV, or RAB7A^High^-sEV. (B-C) Representative images of orthotopic MDA-MB-231 tumors (B) and tumor weights at sacrifice (C). (D-E) Representative IHC images and quantitative analyses of Fascin, p-paxillin, and PD-L1 in orthotopic tumor tissues (D), together with corresponding IF staining and quantification (E). (F-G) Representative images of excised orthotopic 4T1 tumors (F) and tumor weights at sacrifice (G). (H) Tumor growth curves of orthotopic 4T1 tumors in mice treated with PBS, RAB7A^Low^-sEV, or RAB7A^High^-sEV. (I) ssGSEA-based comparison of the CD8^+^ T-cell exhaustion score between tumors treated with RAB7A^Low^-sEV and RAB7A^High^-sEV. (J-K) Representative IHC images and quantification (J), as well as IF staining and quantification (K), of CD4, CD8 and PD-L1 in orthotopic tumor tissues. Statistical significance is indicated as *P < 0.05, **P < 0.01, and ***P < 0.001. IF: immunofluorescence; IHC: Immunohistochemistry; PBS: phosphate buffer saline; PD-L1: programmed cell death ligand 1; TNBC: Triple-negative breast cancer.

**Figure 5:**
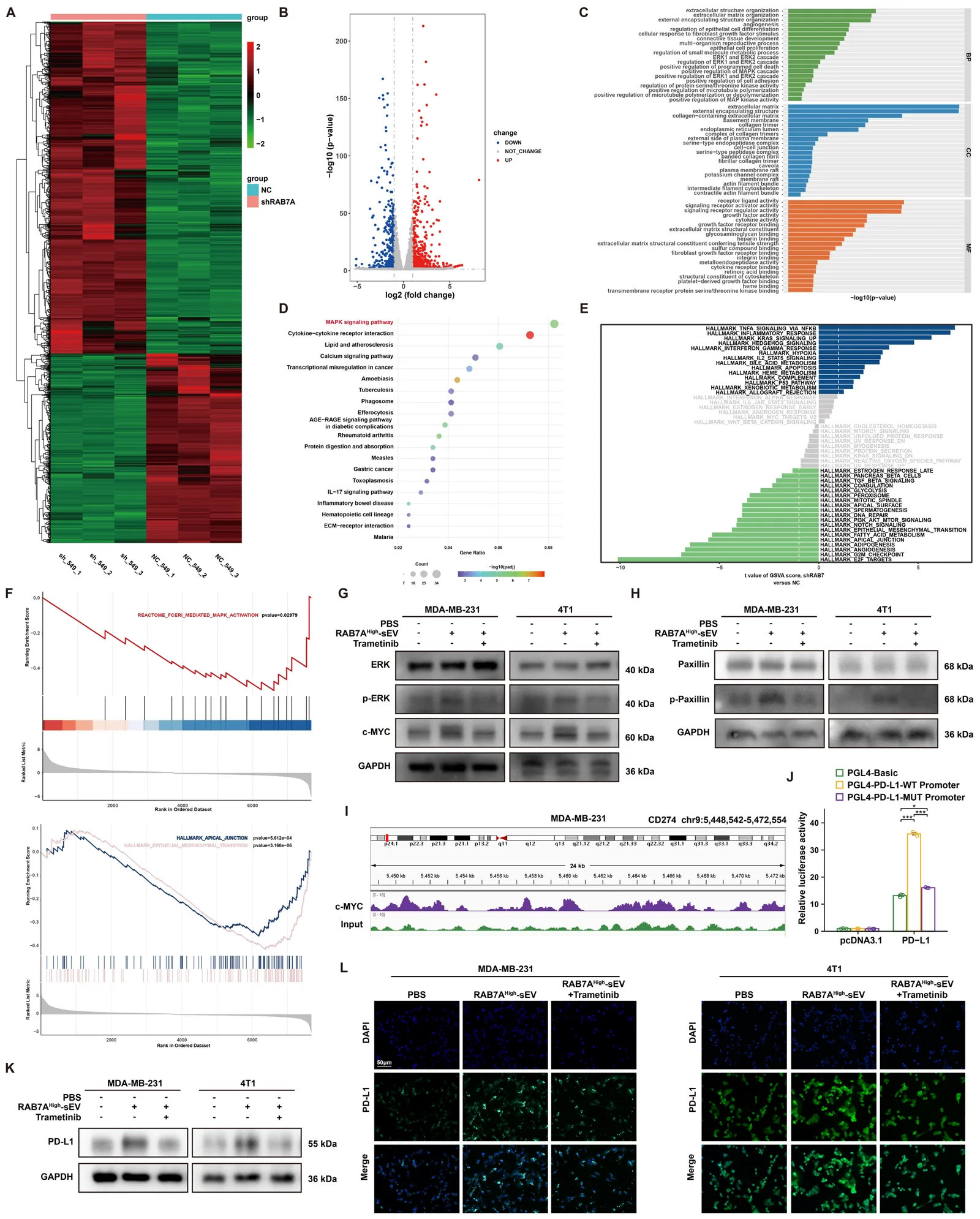
** RAB7A^High^-sEV promotes TNBC cytoskeletal remodeling and upregulates PD-L1 through the ERK/c-MYC pathway.** (A) Heatmap showing differentially expressed genes in control (sh-NC) and RAB7A-knockdown (sh-RAB7A) BT-549 cells. (B) Volcano plot of differentially expressed genes between sh-NC and sh-RAB7A BT-549 cells. Red and blue dots indicate upregulated and downregulated genes, respectively. (C) GO enrichment analysis of differentially expressed genes. (D) KEGG analysis shows that differentially expressed genes were enriched in MAPK signaling pathway. (E) GSVA showing differentially enriched hallmark pathways between the sh-NC and sh-RAB7A groups. (F) GSEA demonstrating enrichment gene sets in sh-NC relative to sh-RAB7A BT-549 cells. (G) Western blot analysis of ERK, p-ERK, and c-MYC in MDA-MB-231 and 4T1 cells treated with PBS, RAB7A^High^-sEV or RAB7A^High^-sEV combined with the ERK inhibitor trametinib. (H) Western blot analysis of paxillin and p-paxillin in MDA-MB-231 and 4T1 cells following the indicated treatments. (I) Genome-browser tracks showing c-MYC occupancy at the CD274 (PD-L1) locus in MDA-MB-231 cells, based on CHIP-seq data. (J) Dual-luciferase reporter assay assessing the transcriptional activity of the wild-type and mutant PD-L1 promoter constructs in MDA-MB-231 cells. (K) Western blot analysis of PD-L1 expression in MDA-MB-231 and 4T1 cells under the indicated treatment conditions. GAPDH was used as the loading control. (L) Representative immunofluorescence images of PD-L1 (green) in MDA-MB-231 and 4T1 cells after the indicated treatments; nuclei were counterstained with DAPI (blue). Statistical significance is indicated as *P < 0.05, **P < 0.01, and ***P < 0.001. CHIP-seq: chromatin immunoprecipitation sequencing; GO: Gene Ontology; GSEA: Gene set enrichment analysis; GSVA: Gene set variation analysis; KEGG: Kyoto Encyclopedia of Genes and Genomes; PBS: phosphate buffer saline.

**Figure 6:**
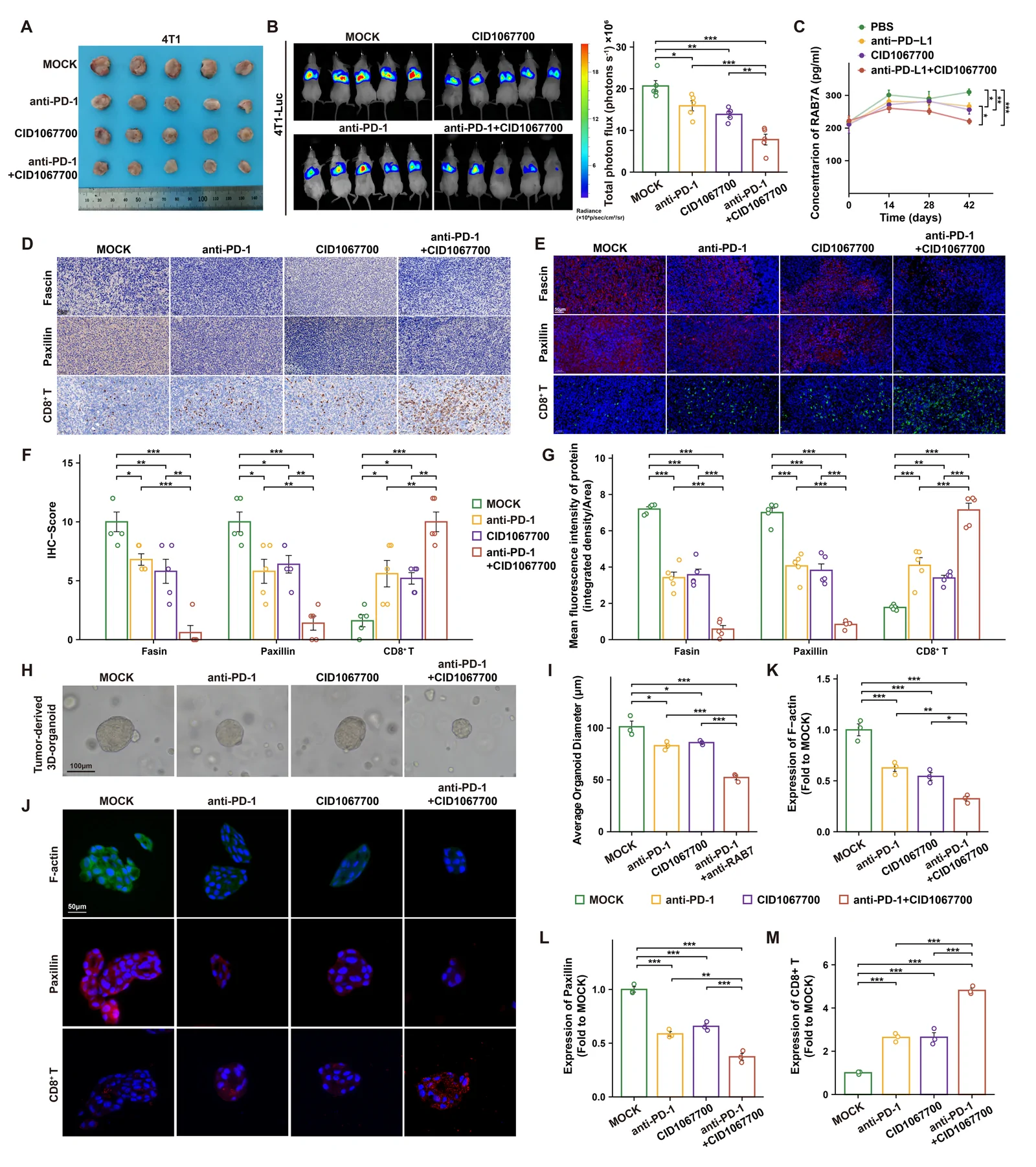
** Combined anti-PD-1 and CID1067700 treatment suppresses TNBC progression and remodels the tumor immune microenvironment.** (A) Representative images of excised orthotopic 4T1 tumors from mice treated with vehicle control (MOCK), anti-PD-1 antibody, CID1067700, or their combination. (B) Representative bioluminescence images and quantification of total photon flux in the experimental lung-metastasis model following the indicated treatments. (C) RAB7A concentrations within plasma-derived sEV in each treatment group. (D-E) Representative IHC images (D) and IF images (E) of Fascin, paxillin, and CD8 in orthotopic tumor tissues. (F-G) Quantification of IHC scores (F) and mean IF fluorescence intensities (G) for Fascin, paxillin, and CD8 staining. (H) Representative bright-field images of PDOs treated as indicated. (I) Quantification of average PDO diameter. (J) Representative IF images of F-actin, paxillin, and CD8 in PDOs following the indicated treatments. (K-M) Quantification of F-actin (K), paxillin (L), and CD8^+^ T-cell (M) staining in PDOs. Statistical significance is indicated as *P < 0.05, **P < 0.01, and ***P < 0.001. IF: immunofluorescence; IHC: Immunohistochemistry; PDO: patient-derived organoids.

**Figure 7:**
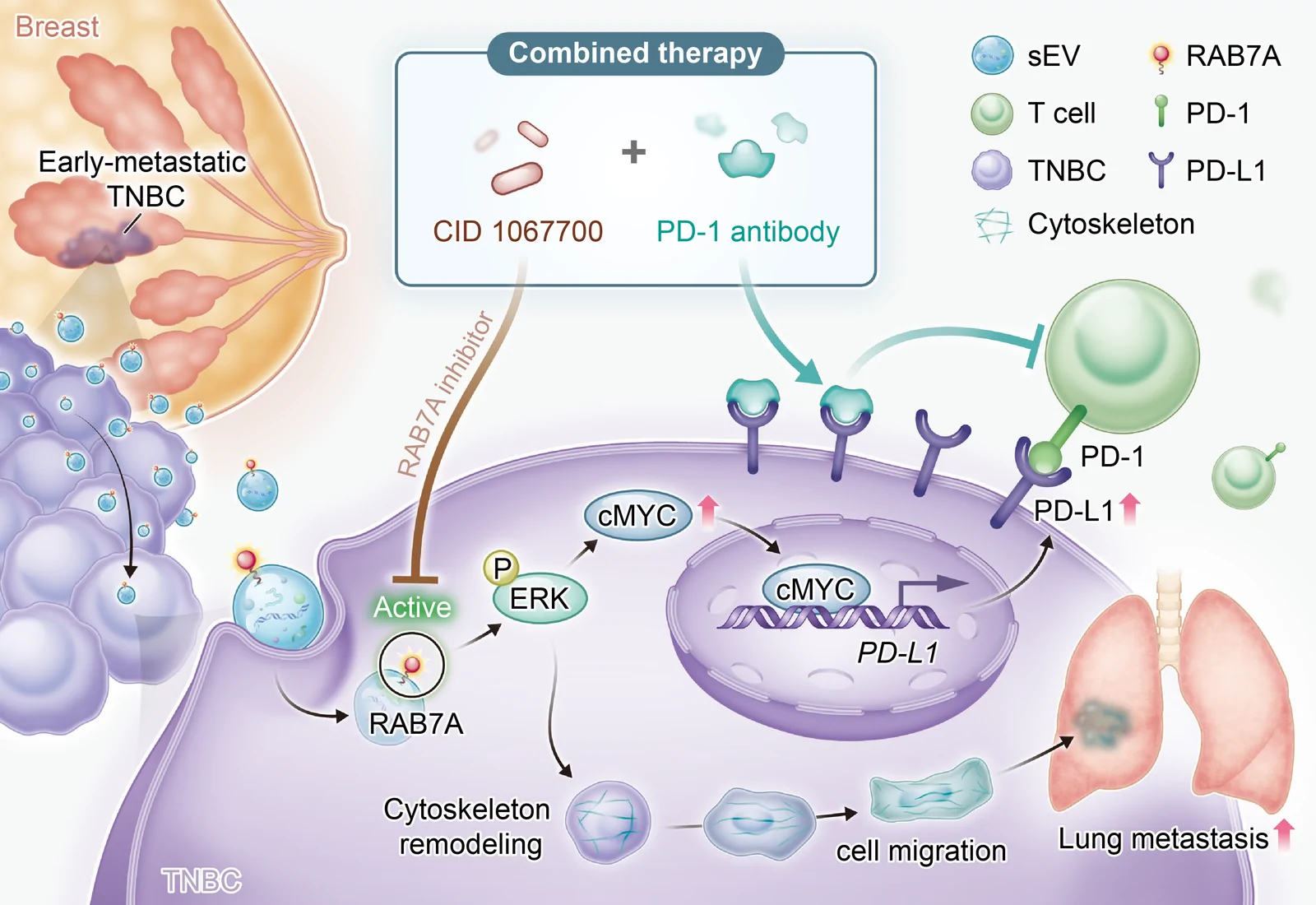
** Working model.** sEVs are derived by tumor cells and released into the microenvironment. Then RAB7A-abundant sEV could be taken up by tumor cells, which upregulates the expression of PD-L1 through ERK/c-MYC pathway and activated cytoskeleton remodeling. As a result, sEV-RAB7A modulates the immunosuppressive microenvironment by suppressing T-cell infiltration and promotes tumor growth and lung metastasis. Blockade of RAB7A can sensitize patients to the immunotherapy response of PD-1, thus inhibiting TNBC metastasis. PD-1: programmed cell death protein-1; TNBC: Triple-negative breast cancer.

## Data Availability

The authors confirm that the data supporting the findings of this study are available within the article and its supplementary materials and further inquiries can be directed to the corresponding authors.

## Abbreviations

ELISA: enzyme linked immunosorbent assay
EMT: epithelial-mesenchymal transition
ER: estrogen receptor
GO: Gene Ontology
GSEA: Gene Set Enrichment Analysis
GSVA: Gene Set Variation Analysis
IF: immunofluorescence
IHC: Immunohistochemistry
KEGG: Kyoto Encyclopedia of Genes and Genomes
OS: overall survival
PD-1: programmed cell death protein-1
PD-L1: programmed cell death ligand 1
PDO: patient-derived organoids
PR: progesterone receptor
sEV: small extracellular vesicles
TIME: tumor immune microenvironment
TNBC: Triple-negative breast cancer
