# Supplementary material for: RAB7A-enriched extracellular vesicles drive early-metastasis and immune evasion in triple-negative breast cancer
Source: Int J Biol Sci. 2026 Sep 18;22(15):8348–63. doi: 10.7150/ijbs.138205 (PMC13685767; doi:10.7150/ijbs.138205)
Supplement: Supplementary file 1 [file ijbsv22p8348s1.pdf]

Supplementary materials

Supplementary Figures

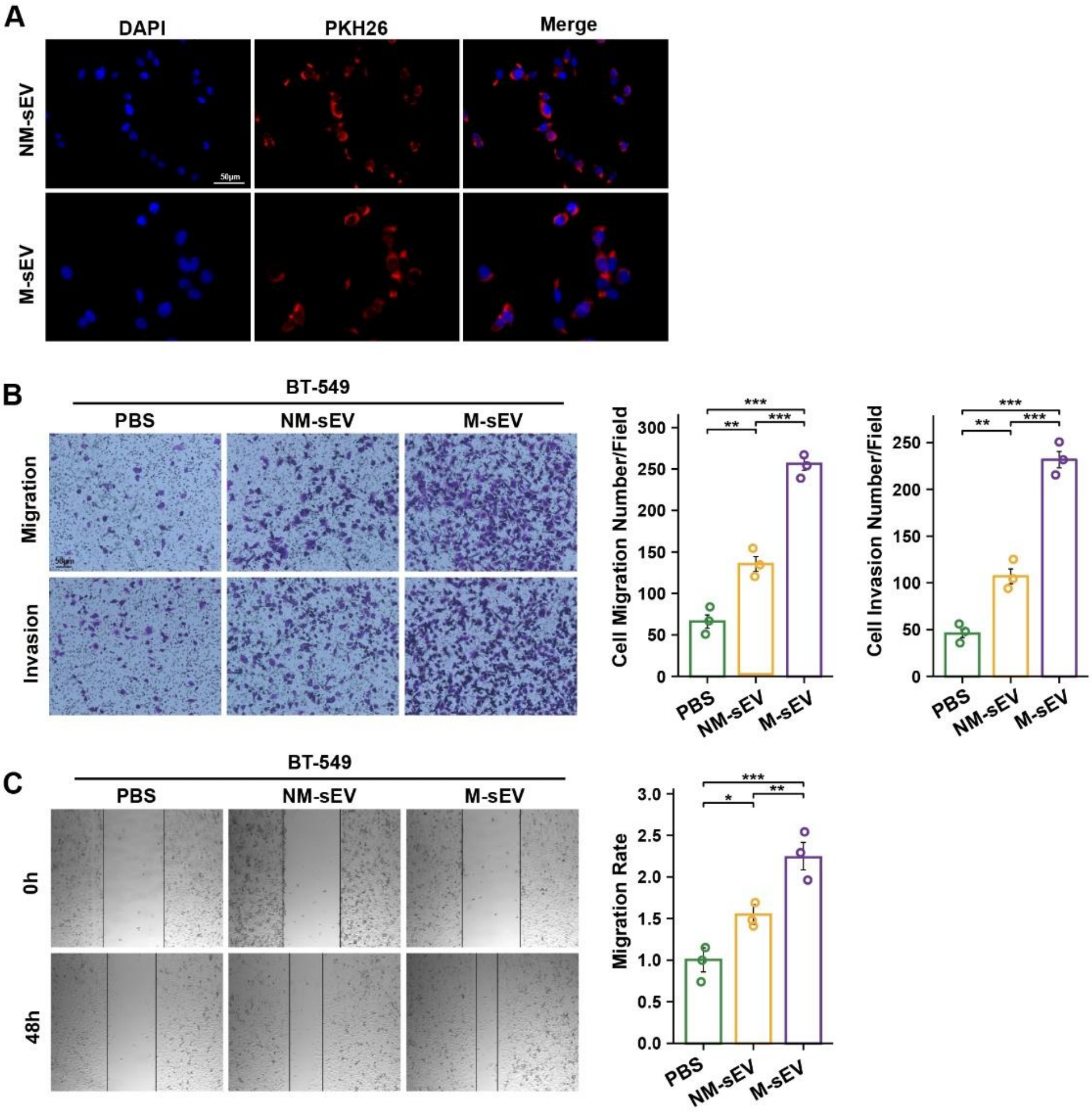

Figure S1. (A) Representative confocal microscopy images showing uptake of PKH26-labeled NM-sEV and M- sEV by MDA-MB-231 cells. (B) Representative images of transwell assays and statistical analysis of BT-549 cell treated with PBS, NM-sEV or M-sEV. (C) Representative images of wound healing assays and statistical analysis of BT-549 cell treated with PBS, NM-sEV or M-sEV. $^*P < 0.05$, $^{**}P < 0.01$, $^{***}P < 0.001$. M-sEV: EVs that were extracted from breast cancer patients suffering metastasis; NM-sEV: EVs that were extracted from breast cancer patients without metastasis.

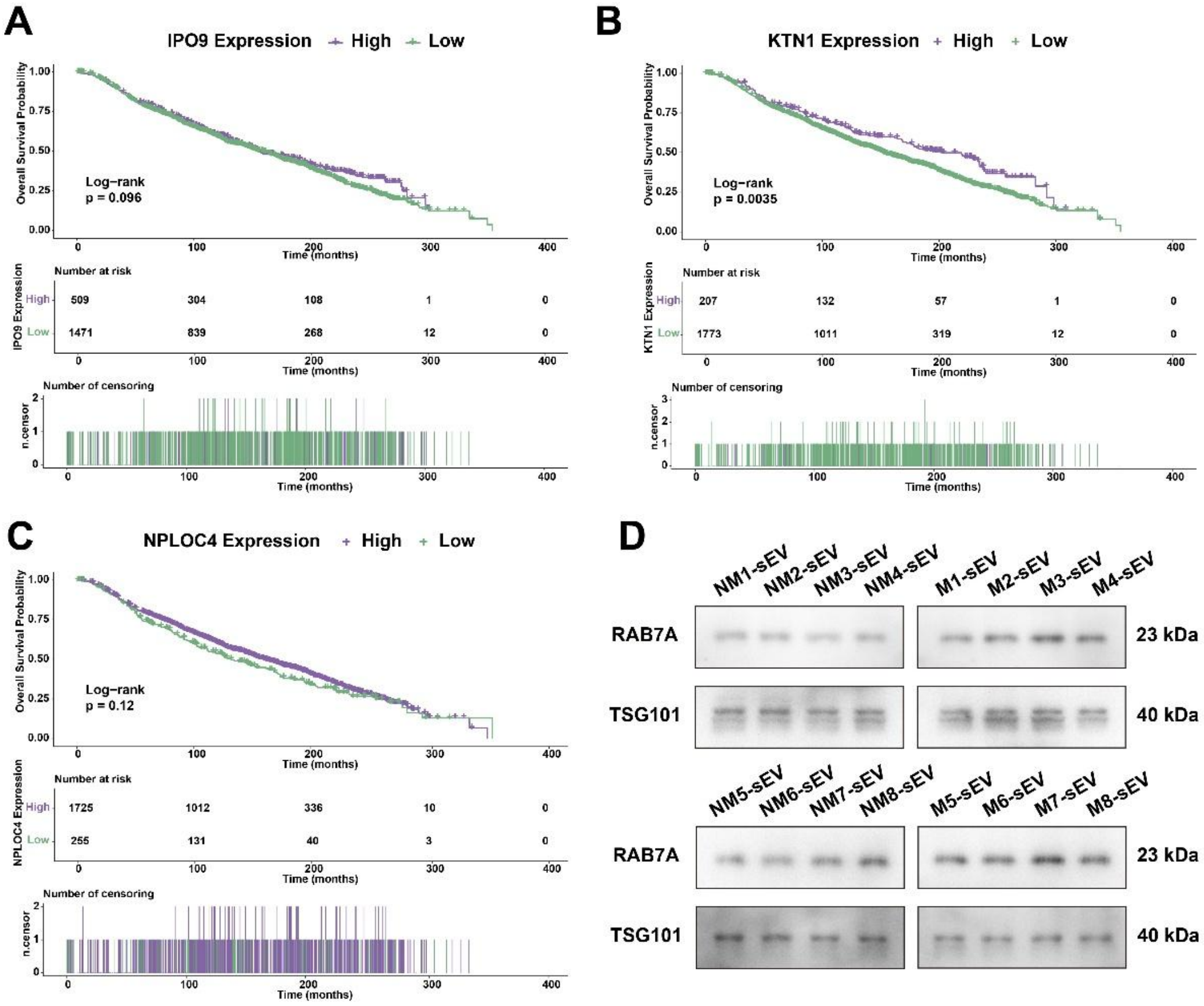

Figure S2. (A) Kaplan-Meier curve showing no significant association between NPLOC4 expression and patient overall survival (p = 0.12). (B) Kaplan-Meier curve showing no significant association between IPO9 expression and patient overall survival (p = 0.096). (C) Kaplan-Meier curve indicating that high expression of KTN1 is associated with significantly longer overall survival (p = 0.0035). (D) Western blot analysis of RAB7A in plasma sEV from patients with NM-TNBC and M-TNBC; TSG101 was used as an sEV marker.

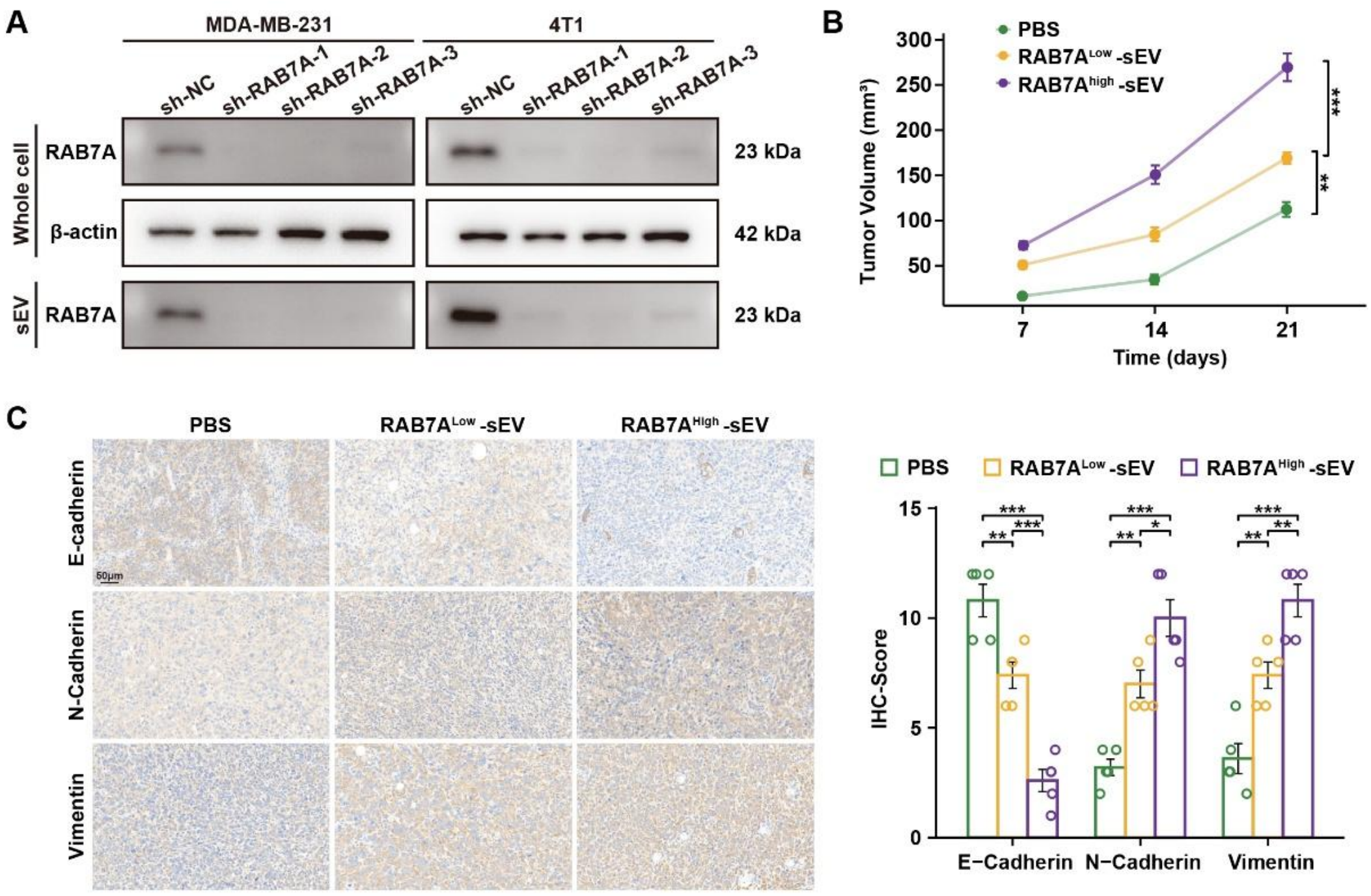

Figure S3. (A) Western blot analysis of RAB7A expression in whole-cell lysates and sEVs isolated from MDA-MB-231 and 4T1 cells expressing sh-NC or three independent sh-RAB7A constructs. β-actin was used as a loading control for whole-cell lysates. (B) Growth curves of orthotopic tumors in mice treated with PBS, RAB7A^{Low}-sEV, or RAB7A^{High}-sEV. (C) Representative immunohistochemical images and H-scores of E-cadherin, N-cadherin, and vimentin in orthotopic tumor tissues. **P < 0.01, ***P < 0.001.

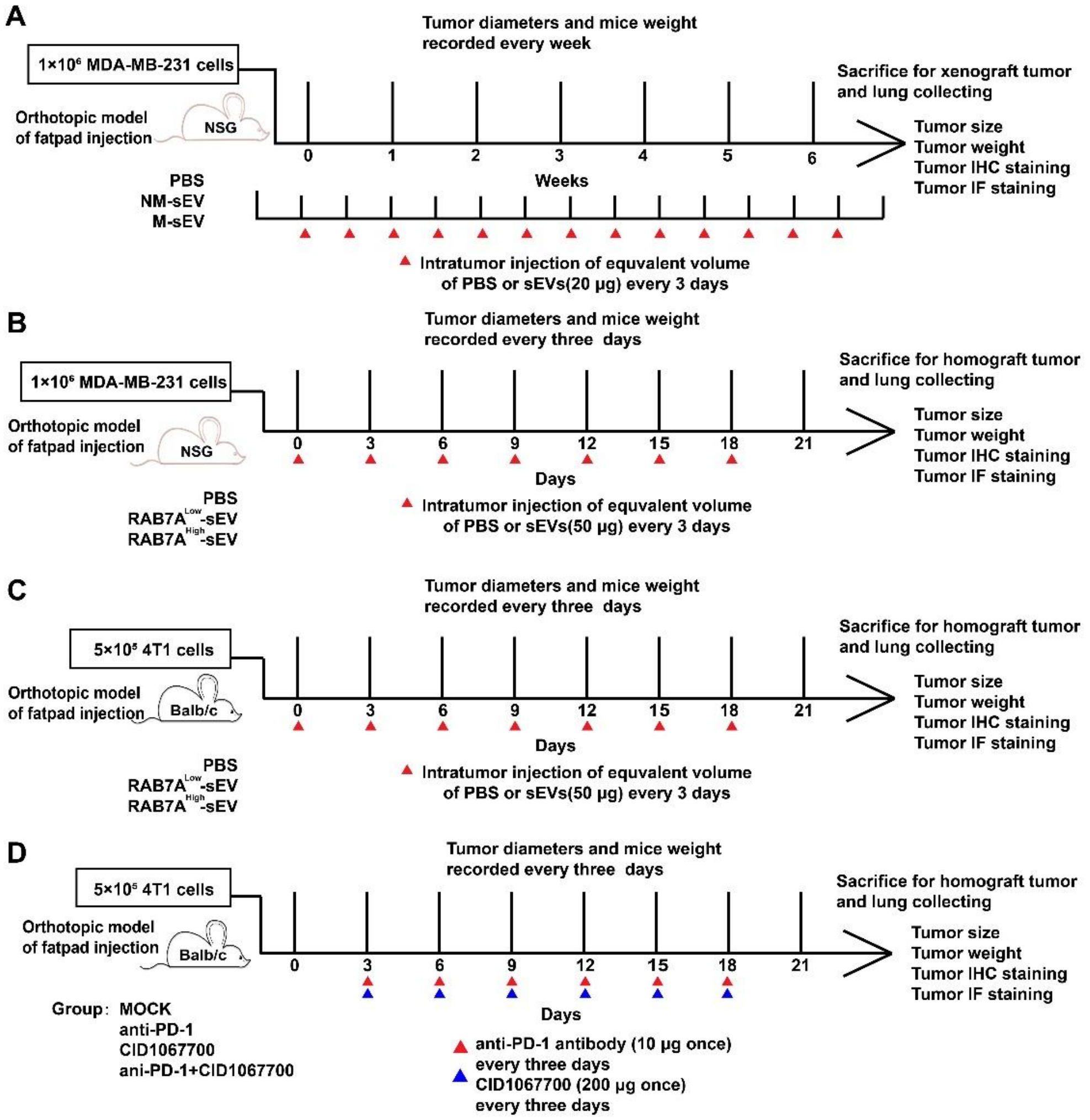

Figure S4. Schematic diagrams of the orthotopic models. (A) Suspensions of 1×10^6 MDA-MB-231 cells were injected to each mouse, PBS, NM-sEVs or M-sEVs were injected subcutaneously into NSG mice every 3 days. (B) Suspensions of 1×10^6 MDA-MB-231 cells per mouse were implanted to establish orthotopic tumors in NSG mice. PBS, $RAB7A^{Low}$-sEV or $RAB7A^{High}$-sEV were injected subcutaneously every 3 days. (C) Suspensions of 5×10^4 4T1 cells per mouse was injected in BALB/c mice. PBS, $RAB7A^{Low}$-sEV or $RAB7A^{High}$-sEV were injected subcutaneously every 3 days. (D) Suspensions of 5×10^4 4T1 cells per mouse were injected into BALB/c mice, and anti-PD-1 antibody (10 µg once) and CID1067700 (200 µg once) were injected every 3 days. The tumor weight of each mouse was measured at the time of sacrifice. Tumor tissue samples were subjected to IHC and IF analyses, respectively. Moreover, the tumors in (C) were subjected to bulk RNA sequencing. M-sEV: sEV that were extracted from breast cancer patients suffering early-metastasis; NM-sEV: sEV that were extracted from breast cancer patients without metastasis; HE: IHC: immunohistochemistry.

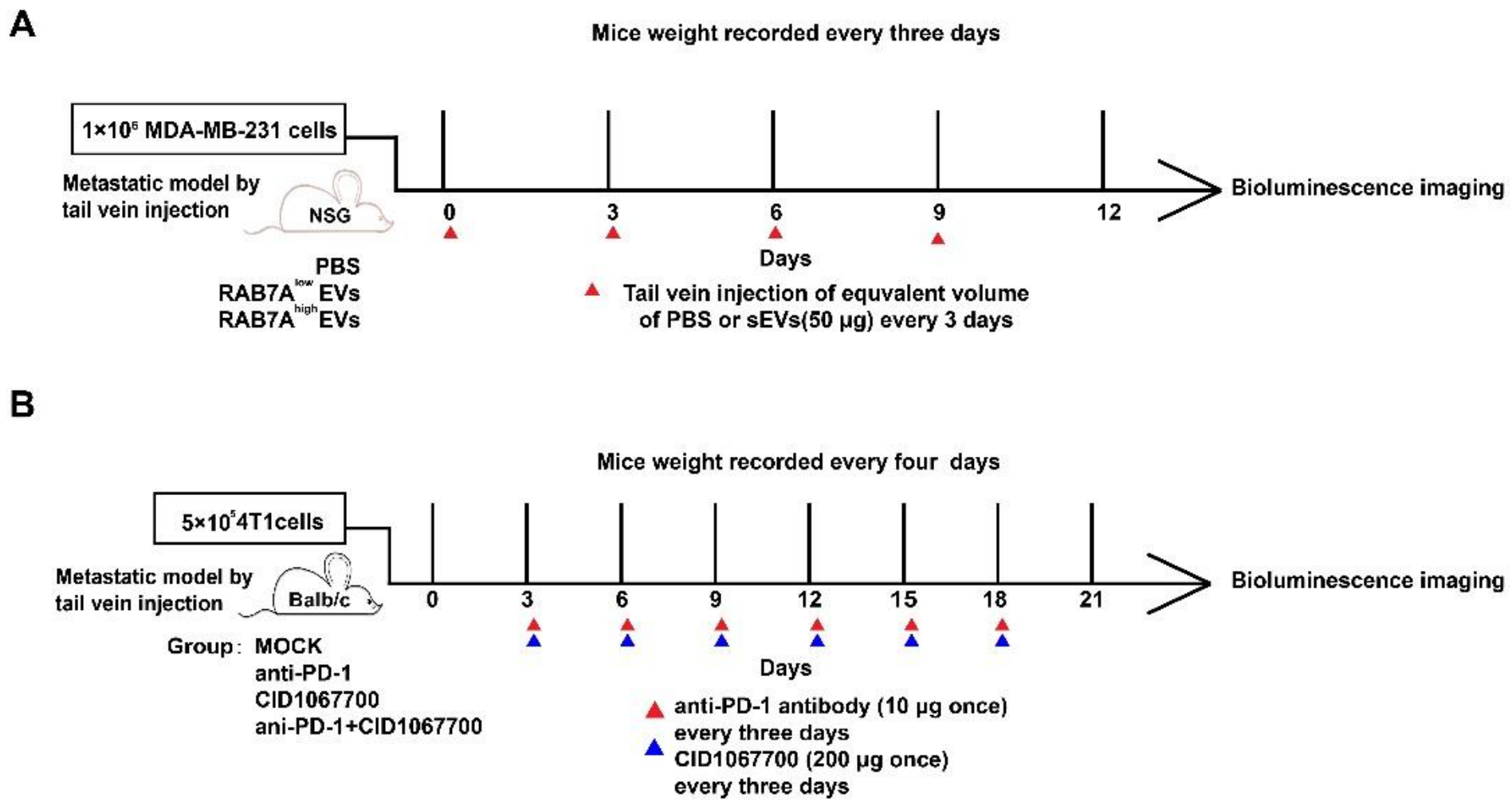

Figure S5. Schematic diagrams of the metastatic models. (A) A total of 1×10^6 MDA-MB-231 cells per mouse were injected by tail vein to establish metastatic models in NGS mice, PBS, $RAB7A^{Low}$-sEV or $RAB7A^{High}$-sEV were injected subcutaneously every 3 days. (B) 4T1 cells (5×10^4 cells/mouse) were subcutaneously injected into BALB/c mice, and anti-PD-1 antibody (10 µg once) and CID1067700 (200 µg once) were injected every three days. Lung samples were measured with bioluminescence imagine, respectively.

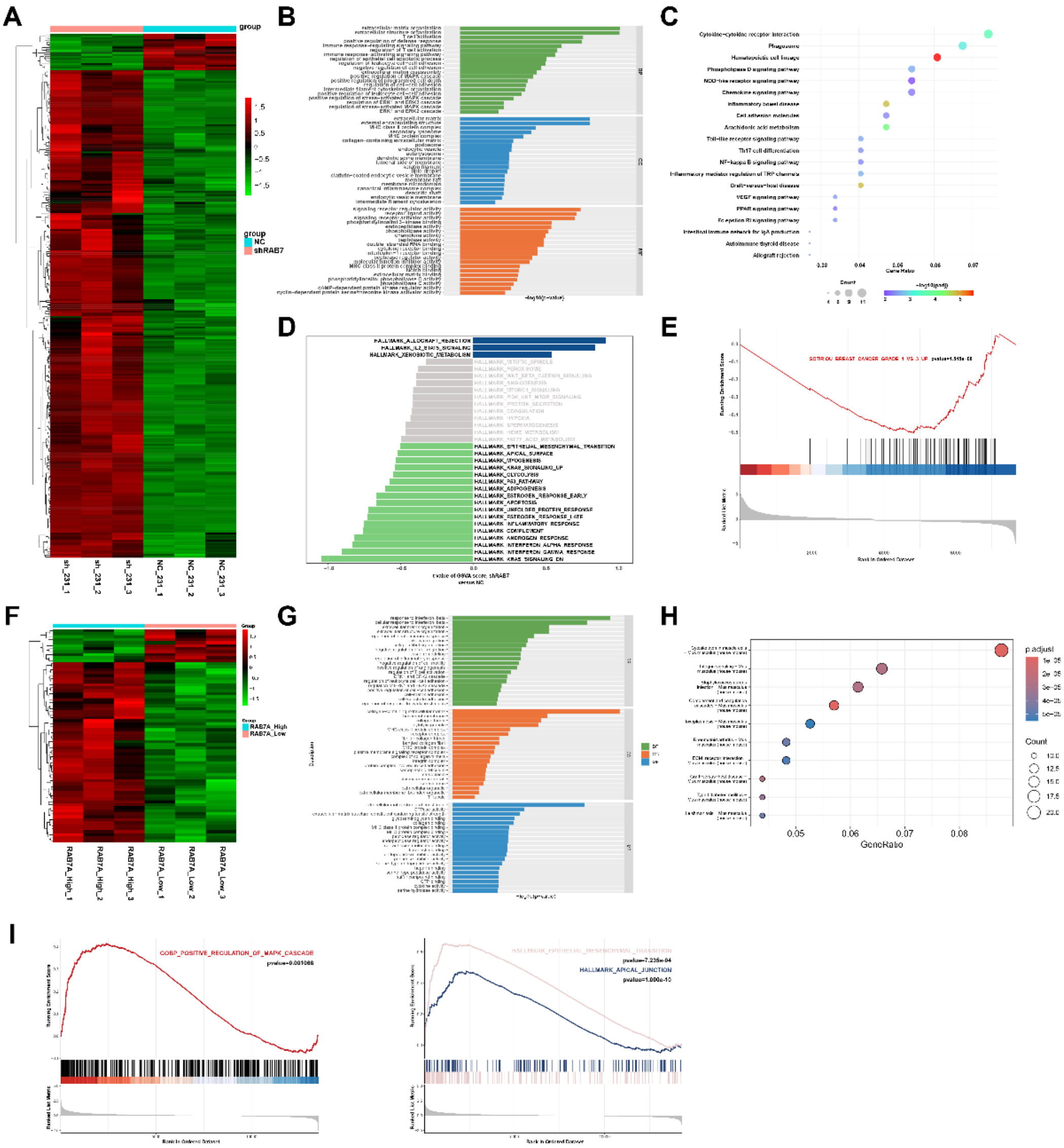

Figure S6. Transcriptomic Analysis of RAB7A-Knockdown in MDA-MB-231 Cells and sEV-treated orthotopic 4T1 tumors. (A) Heatmap of hierarchical clustering based on the expression of top significant DEGs between control (shNC) and RAB7-knockdown (shRAB7) MDA-MB-231 cells. (B) GO biological process enrichment analysis for DEGs from RAB7-knockdown MDA-MB-231 cells. (C) KEGG pathway enrichment analysis for DEGs from RAB7-knockdown MDA-MB-231 cells. (D) Bar plot of GSVA scores for selected Hallmark gene sets in control and RAB7-knockdown MDA-MB-231 cells. (E) GSEA plot showing enrichment of a breast cancer stage grade signature in control versus RAB7-knockdown MDA-MB-231 cells. (F) Heatmap of hierarchical clustering based on the expression of top significant DEGs between orthotopic 4T1 tumors treated with RAB7A^{High}-sEV or RAB7A^{Low}-sEV. (G) GO biological process enrichment analysis for DEGs from orthotopic tumors. (H) KEGG analysis of DEGs from orthotopic 4T1 tumors treated with RAB7A^{High}-sEV or RAB7A^{Low}-sEV. (I) GSEA pathways enrichment analysis of DEGs from tumors treated with RAB7A^{High}-sEV or RAB7A^{Low}-sEV. GO: Gene Ontology; KEGG: Kyoto Encyclopedia of Genes and Genomes; GSVA: Gene set variation analysis; GSEA: Gene set enrichment analysis.

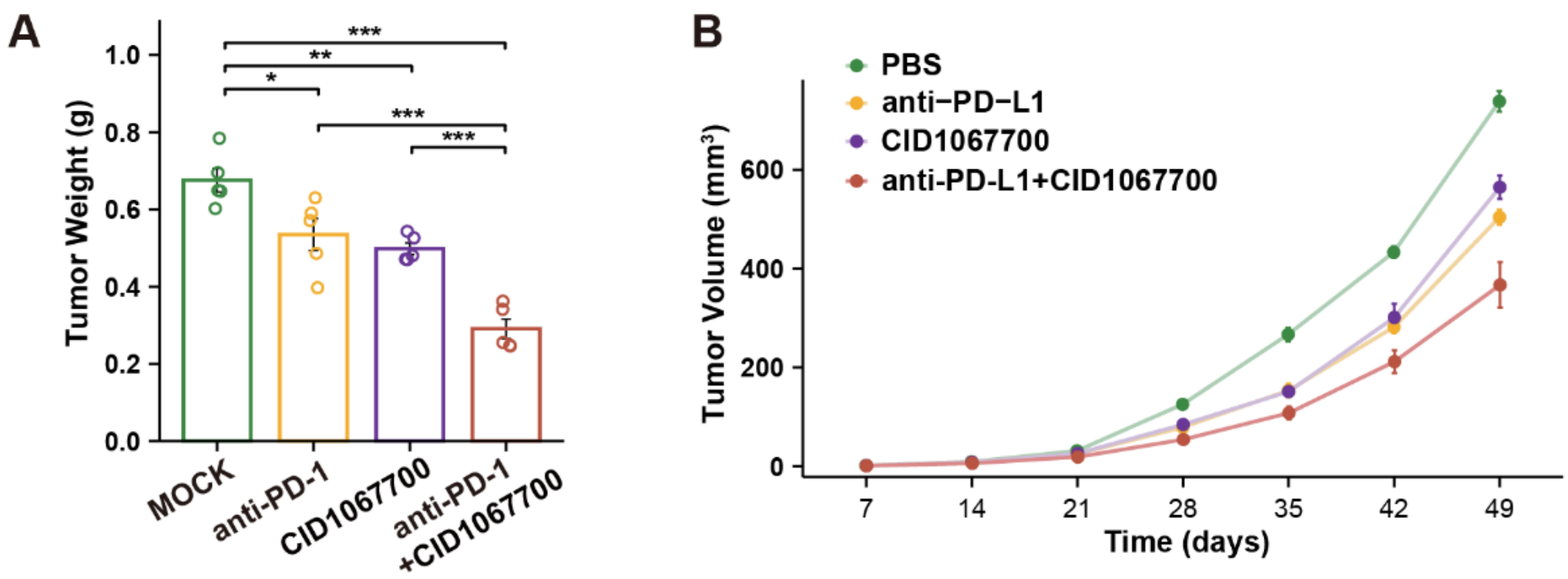

Figure. S7 (A-B) Orthotopic tumor weights (A) and growth curves (B) of the mice subjected to different treatments are shown. The injection of the anti-PD-1 antibody (10 μg once) and/or CID1067700 (200 μg once) was scheduled every 3 days. **P* < 0.05, ***P* < 0.01, ****P* < 0.001.

Supplementary Tables

Table S1. Primary antibodies for western blot, immunofluorescence and immunohistochemistry.

Protein	Concentration Western blot	Concentration IF	Concentration IHC	Specificity	Company Lot No.
CD4	/	1:100	1:100	Rat anti-mouse	Novus Biologicals Cat#NBP1-19371
CD8	/	1:100	1:100	Rat anti-mouse	Novus Biologicals Cat#NBP1-49045
PD-L1	/	1: 100	/	Rat anti-mouse	Novus Biologicals Cat# NBP1-76769
Phalloidin	/	1:100	/	/	CST Cat#8878
Fascin	/	1:50	/	Mouse anti-mouse or human	CST Cat#54545
Fascin	/	/	1:100	Mouse anti-mouse or human	CST Ca#99978
Paxillin	1:1000	1:100	1:100	Rabbit anti-mouse or human	CST Cat#50195
Phospho-paxillin	1:1000	1:200	1:200	Rabbit anti-mouse or human	CST Cat#69363
RAB7A	1:1000	/	/	Rat anti- human	CST Cat#9272
GAPDH	1:1000	/	/	Rat anti-mouse or human	CST Cat#5174

PD-L1: Programmed death ligand 1; IF: Immunofluorescence; IHC: Immunohistochemistry.

Table S2. Clinical parameters and pathological information applied in this study for RAB7 analysis.

NO.	Age	Menopausal status	Second primary cancer	cT	cN	Pathology	Grade	HER-2	Ki-67
Case1	≤50	Postmenopausal	No	T2	N0	IDC	III	Low	50%
Case2	>50	Postmenopausal	No	T3	N+	IDC	III	Negative	85%
Case3	>50	Postmenopausal	No	T3	N0	IDC	III	Negative	90%
Case4	≤50	Premenopausal	No	T2	N+	IDC	III	Low	60%
Case5	>50	Postmenopausal	No	T3	N0	IDC	III	Negative	90%
Case6	≤50	Premenopausal	No	T1	N+	IDC	III	Low	50%
Case7	≤50	Premenopausal	No	T2	N0	IDC	III	Negative	70%
Case8	>50	Postmenopausal	No	T2	N0	IDC	II	Low	40%
Case9	≤50	Premenopausal	No	T2	N0	IDC	III	Negative	70%
Case10	>50	Postmenopausal	No	T1	N0	IDC	II	Low	20%
Case11	>50	Postmenopausal	No	T1	N0	IDC	II	Negative	15%
Case12	>50	Postmenopausal	No	T1	N0	IDC	II	Negative	80%
Case13	>50	Postmenopausal	No	T2	N0	IDC	II	Negative	60%
Case14	>50	Postmenopausal	No	T2	N0	IDC	II	Negative	20%
Case15	≤50	Premenopausal	No	T3	N+	IDC	III	Negative	80%
Case16	≤50	Premenopausal	No	T2	N0	IDC	II	Low	5%

IDC: invasive ductal carcinoma, HER-2: Human epidermal growth factor receptor-2

Supplementary methods

sEV isolation

For sEV isolation from cell culture, TNBC cells were grown in 10-cm dishes in serum-free basal medium for 48 hours. sEVs were subsequently isolated from the conditioned medium via ultracentrifugation. Briefly, the medium was sequentially centrifuged at 300 × g for 10 minutes and 2,000 × g for 20 minutes to remove cells and debris. The supernatant was then centrifuged at 10,000 × g at 4°C for 30 minutes to eliminate large vesicles. Finally, the sample was subjected to ultracentrifugation at 100,000 × g at 4°C for 2 hours. The resulting pellet was washed once by resuspension in phosphate-buffered saline (PBS) and a final ultracentrifugation step at 150,000 × g at 4°C for 2 hours. The purified sEV pellet was resuspended in PBS for downstream applications.

For sEV isolation from patient plasma, whole blood was collected from fasted donors into K_2-EDTA tubes (BD Biosciences, San Jose, CA, USA). Plasma was separated by two consecutive centrifugation steps at 500 × g for 15 minutes at room temperature to remove blood cells. The plasma was then centrifuged at 2,500 × g for 15 minutes to pellet platelets and large debris. The clarified plasma was aliquoted and stored at -80°C, with freeze-thaw cycles limited to a maximum of one. Prior to sEV isolation, thawed plasma was centrifuged at 10,000 × g at 4°C for 30 minutes to remove apoptotic bodies and large extracellular vesicles. sEVs were subsequently purified from the pre-cleared plasma using an EVssupur size exclusion chromatography column (ES914, ECHO BIOTECH, CN) according to the manufacturer's protocol. Briefly, 1 mL of plasma was loaded onto the column, and ten sequential 0.5 mL fractions were eluted with PBS. Fractions 4 to 6, containing the sEV-enriched eluate, were pooled for subsequent analysis.
